# Supplementary material for: Retrograde transport of TrkB-containing autophagosomes via the adaptor AP-2 mediates neuronal complexity and prevents neurodegeneration
Source: Nat Commun. 2017 Apr 7;8:14819. doi: 10.1038/ncomms14819 (PMC5385568; doi:10.1038/ncomms14819)
Supplement: Supplementary Information — Supplementary Figures and Supplementary Tables. [file ncomms14819-s1.pdf]

Supplementary Table 1. Antibodies used in this study

Antigen	clone/ID	Host	Dilution			Source	Order nr.
			IF	WB	IP		
Akt	-	rabbit	-	1:1000	-	Cell signalling	9272
Amphiphysin1	Amphy#3	mouse	-	1:1000	-	DeCamilli's lab	-
AP-1 gamma adaptin	88	mouse	-	1:1000	-	BD Transduction	610386
AP-2 alpha adaptin	8	mouse	1:200	1:1000	-	BD Transduction	610502
AP-2 alpha adaptin	AC1M11	mouse	-	1:1000	-	Life Sciences	MA3-061
AP-2 alpha adaptin	AP-6	mouse	1:400	-	-	Haucke's lab	-
AP-2 beta adaptin	-	goat	-	-	10µg/ sample	Santa Cruz Biotechnology	SC6425
AP-2 beta adaptin	H-300	rabbit	-	1:1000	-	Santa Cruz Biotechnology	SC10762 (lot # E1304)
AP-3 µ3	p47	mouse	-	1:500	-	BD Transduction	discontinued
AP180	LP2D11	mouse	-	1:3333	-	Zhang/De Camilli's lab	-
BDNF	N-20	rabbit	-	1:200	-	Santa Cruz	sc-546
cleaved Caspase (Asp175)3	-	rabbit	-	1:1000	-	Cell Signalling	9661
CHC	TD1	mouse	-	1:500	-	Haucke's Lab	-
c-Myc	9E10	mouse	1:400	-	-	Sigma	M5546
Dynamin1	Dynamin1 #5	mouse	-	1:500	-	De Camilli lab	-
GFP	-	rabbit	1:10000	-	-	Abcam	ab6556

GFP	-	rabbit	1:1000	1:1000	-	MBL	598
Grb2	3F2	mouse	1:200	-	-	MBL	MS-20-3
HA	-	rabbit	1:400	-	-	Cayman	162200
His- tag	27E8	mouse	-	1:1000	-	Cell Signaling Technology	2366S
HSC70	-	mouse	-	1:5000	-	Affinity bioreagents	-
Hsc70	-	rat	-	1:5000	-	Enzo	SPA-815 (lot# 42)
IgG	-	goat	-	-	10µg/ sample	Sigma-Aldrich	I5256
ITSN1	mAC+ linker	rabbit	-	1:1000	-	Shupliakov's lab	-
KCC2	-	rabbit	1:250	-	-	Jentsch's lab	-
LC3b	4E12	mouse	1:100	-	-	MBL	M152-3
LC3b	-	rabbit	1:250	1:500	-	Novus biochemical	NB600-1384
mTOR	7C10	rabbit	-	1:1000	-	Cell Signalling	2983
p75NGF	-	rabbit	1:500	-	-	Abcam	ab8874
p150^{Glued}	-	goat	1:100	-	-	Abcam	ab11806
p150^{Glued}	1	mouse	-	1:1000	-	BD Transduction	610474 (lot# 07312)
pmTOR Ser2448	D9C2	rabbit	-	1:1000	-		5536
pRaptor Ser792	-	rabbit	-	1:1000	-	Cell Signalling	2083
pS6 Ser235/236	-	rabbit	-	1:1000	-	Cell Signalling	4856
pTrkB816	-	rabbit	1:500	-	-	Abcam	75173
p75NGF	-	rabbit	1:500	1:1000		Abcam	8874
p62	-	Guinea-pig	1:250	-	-	Progen	GP62-C
Rab7	-	goat	1:250	-	-	GeneTex	discontinued
S6	-		-	1:1000	-	Cell Signalling	2217

Stonin2	2424.5	rabbit	-	1:800	-	Haucke’s Lab	-
Synaptophysin	7.2	mouse	-	1:2000	-	Synaptic Systems	101011
Synaptotagmin 1	41.1	mouse	-	1:500	-	Jahn’s lab	-
TrkB	extracell. domain	rabbit	1:100-1:200	1:500-1:1000	-	Alomone Labs	ANT-019
Tubulin	B-5-1-2	mouse		1:5000	-	Sigma	T5168
Tubb3	-	rabbit	1:2000	-	-	Covance	PRB-435P

Supplementary Table 2. Primers used in qPCR.

Gene description	forward primer	reverse primer
BDNF	TCATACTTCGGTTGCATGAAGG	AGACCTCTCGAACCTGCCC
GAPDH	AACTTTGGCATTGTGGAAGG	ACACATTGGGGGTAGGAACA

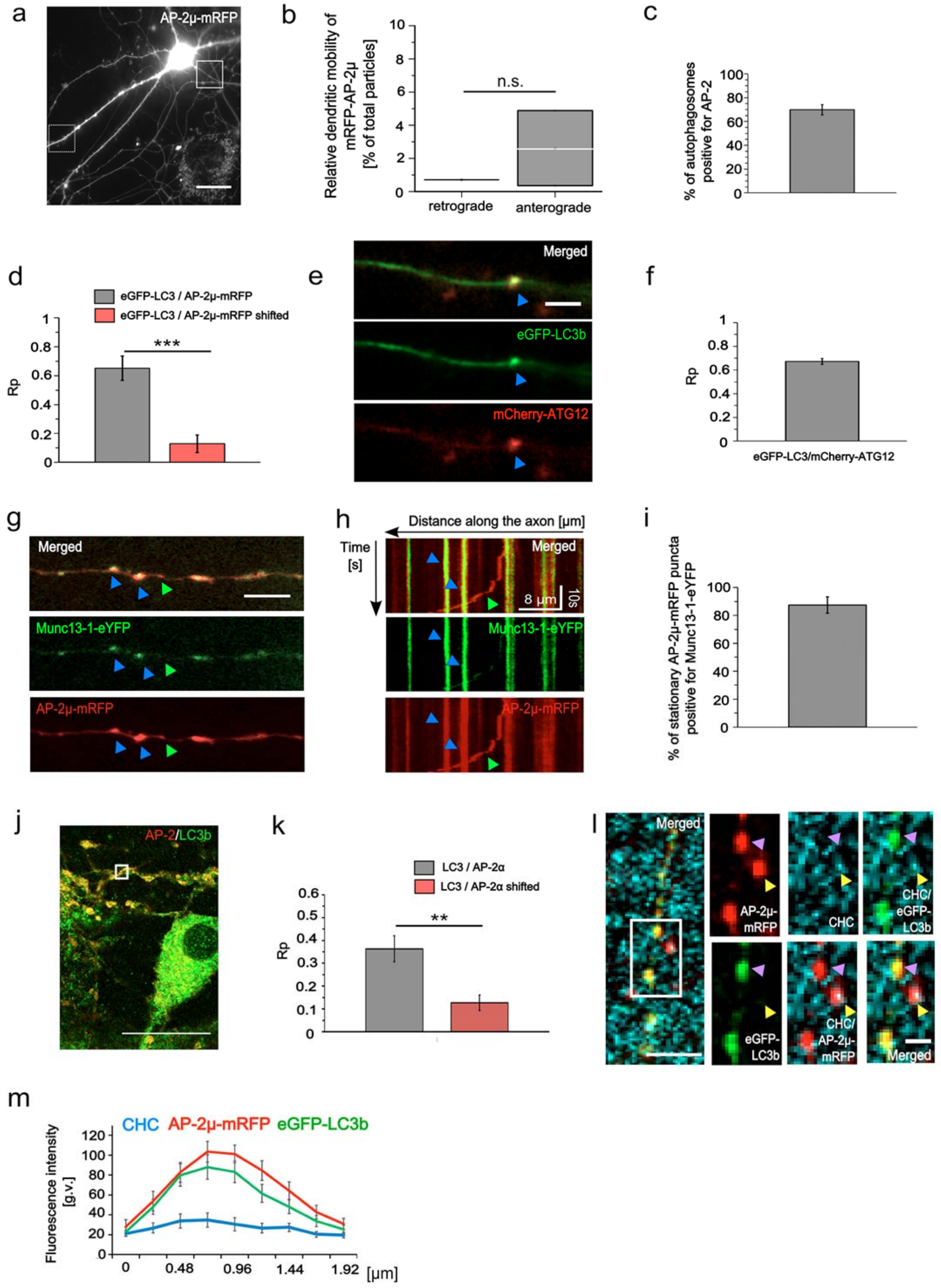

Supplementary Fig. 1. Dynamics of mRFP-AP-2μ transport in dendrites. (**a**) Representative image of a neuron, expressing mRFP-AP-2μ construct. White rectangular boxes indicate the images represented in Fig.1. Scale bar, 20 μm. (**b**) Percentage of retrograde (0.71±0.01%) and anterograde AP-2μ-mRFP puncta in dendrites (2.60±1.17%, n=3 independent experiments). n.s.,

non significant. (**c**) The relative number of eGFP-LC3b puncta colocalizing with AP-2μRFP in axons of cultured neurons (69.8±4.4% from total LC3b-eGFP puncta, n=3 independent experiments). (**d**) Non-random colocalization of AP-2μ-mRFP and eGFP-LC3b puncta in neurons. Pearson's correlation coefficient of Rp=0.65±0.08 indicates the high degree of colocalization between AP-2μ-mRFP and eGFP-LC3b puncta. To exclude the possibility of random colocalization we repeated the analysis after shifting the green (eGFP-LC3b) channel over a distance of 10 pixels relative to the red (AP-2μ-mRFP) channel. The Rp calculated from shifted images (red bar) was significantly lower (Rp=0.13±0.06) compared to non-shifted images (p<.000). (**e,f**) Representative images (**e**) and bar diagram (**f**), revealing the colocalization of eGFP-LC3b with ATG12-mCherry in axons of control cultured neurons (Rp: 0.67±0.02). Rp was calculated for 28 regions of interest (ROI, 5.1 x 4.3 μm) from n=3 independent experiments. Scale bar in (**e**), 5 μm. (**g**) Confinement of stationary AP-2μ-mRFP puncta to synapses revealed by the presynaptic active zone marker Munc-13-1-eYFP. Scale bar, 5μm. (**h**) Kymographs generated from the data in (**g**). (**i**) Relative number of stationary AP-2μ-mRFP puncta confined to active zones (87.4±5.9% from total AP-2μ-mRFP puncta, n=3 independent experiments). (**j**) STED image of cultured neurons treated with folimycin and immunostained for LC3b and AP-2. Scale bar, 15 μm. White rectangular box indicate the area magnified in Fig. 1h. (**k**) Endogenous AP-2μ partially colocalizes with LC3b in neurons (Rp: 0.36±0.04). Rp was calculated for 18 regions of interest per condition from n=3 independent experiments. **(l, m**). Clathrin heavy chain (CHC) is not enriched on AP-2μ-positive autophagosomes. (**l**) Representative images and (**m**) distribution pattern of CHC fluorescence intensity along AP-2μ-positive autophagomes labeled with eGFP-LC3b (n=3 independent experiments). Scale bars: main, 5 μm, insert, 1μm.

Data in (**b**) are illustrated as box plots as described in Methods. Data in (**c,d,f,ik,m**) and all data reported in the text are mean±SEM.

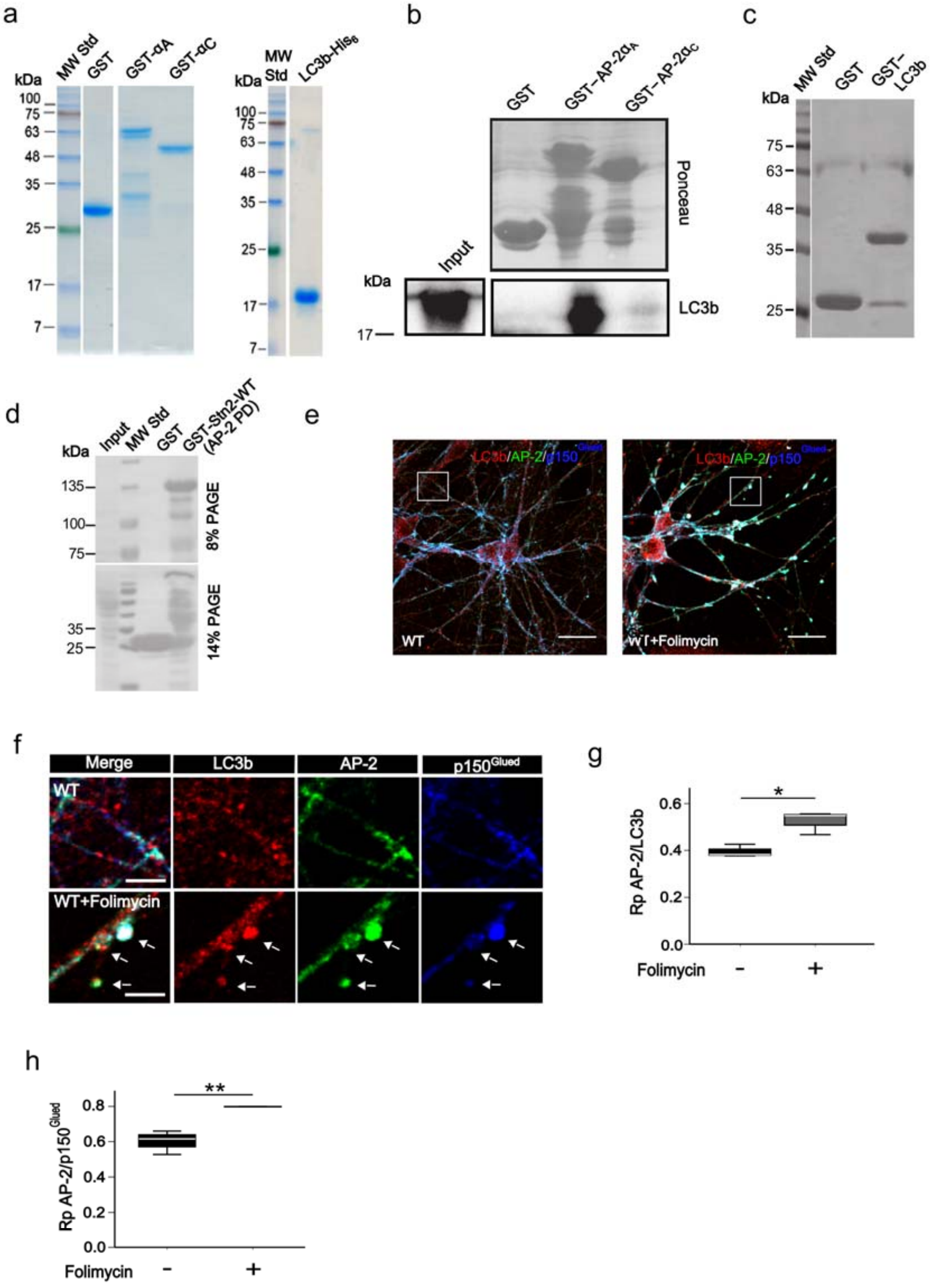

Supplementary Fig. 2. AP-2 colocalizes with LC3b and p150^{Glued} on autophagosomes. (a) Coomassie Blue stained gel of purified proteins used for the experiments shown in Fig. 2a. (**b**) Complex formation of endogenous LC3b with AP-2α_A and AP-2α_C. Upper panel: Ponceau-stained membrane. Lower panel: Immunoblot analysis of material affinity-purified from mouse

brain lysates by GST-AP-2α_A, GST-AP-2α_C or GST taken as a control. AP-2α_A and AP-2α_C co-purify native LC3b present in the lysate, albeit with different efficiency (AP-2α_A > AP-2α_C). Input, 1.5% of lysate added to the assay. (**c**) Coomassie Blue stained gel of purified proteins used for the experiments shown in Fig. 2b. (**d**) Ponceau stained membranes corresponding to the affinity chromatography experiment shown in Fig. 2e. (**e,f)** Representative confocal images of cultured cortico-hippocampal WT or AP-2μ KO neurons left untreated or treated with folimycin and immunostained for LC3b (red), AP-2 (green) and $p150^{Glued}$. Scale bars, (e) 20 μm, (f) 5 μm. White rectangular boxes in (e) indicate the areas magnified in (f). (**g**) Folimycin treatment significantly increases the colocalization of AP-2 with LC3b in cultured cortico-hippocampal neurons (Rp Untreated: 0.40±0.02 versus Rp Folimycin: 0.53±0.03, *p=0.017). Rp was calculated for 60-67 regions of interest (ROI) per condition from 3 independent experiments. (**h**) Folimycin treatment significantly increases the colocalization of AP-2 with $p150^{Glued}$ in cultured cortico-hippocampal neurons (Rp: untreated (UT): 0.60±0.04 versus Rp Folimycin: 0.80±0.001, **p=0.007). Rp was calculated for 60-67 regions of interest (ROI) per condition from 3 independent experiments (n=3).

Data in (g,h) are illustrated as box plots as described in Methods. All data reported are mean±SEM.

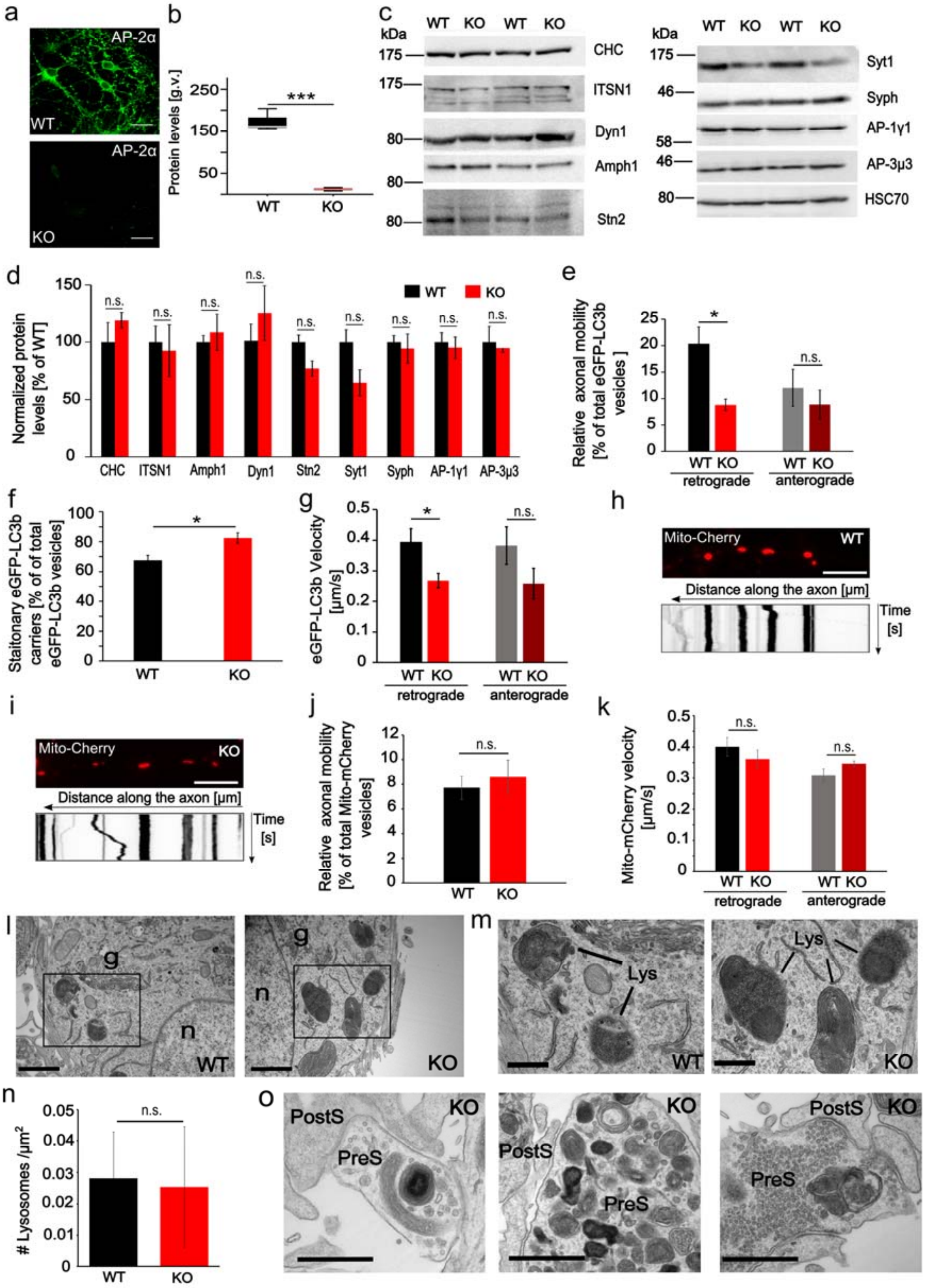

Supplementary Fig. 3. Impaired autophagosome processing in absence of AP-2μ is not a result of alterations in presynaptic and/or endocytic protein levels. (a,b) Loss of AP-2α in

conditional AP-2µ–KO neurons (WT: 173.74±15.19 g.v.; KO: 12.28±2.26 g.v., ***p<0.001, n=3, 30-31 neurons per genotype). g.v., mean grey value of immunofluorescent signal. Scale bar, 20 µm. (**c,d)** Levels of endocytic and presynaptic proteins in brain lysates from p21 WT and neuron-specific AP-2µ KO mice (AP-2$\mu^{lox/lox}$ x Tubα1-Cre). Protein levels in KO condition were normalized to the WT set to 100%. n=3, (**e**) Deletion of AP-2µ significantly decreased the percentage of retrogradely moving autophagosomes (WT: 20.35±3.18%, KO: 8.81± 1.11%, *p=0.01), whereas the number of anterograde carriers is unaltered (WT: 12.04± 3.52%, KO: 8.90± 2.75%, p=0.51, n=4 independent experiments). (**f**) The number of stationary autophagosomes is increased in neurons lacking AP-2µ (WT: 67.65± 4.44%, KO: 82.45± 3.61%, *p=0.04, n=4 independent experiments). (**g**) Deletion of AP-2µ significantly decreased the retrograde autophagosome velocity (WT: 0.39±0.04 µm/sec, KO: 0.27± 0.02 µm/sec, *p=0.048), whereas their anterograde velocity is unaltered (WT: 0.38± 0.06 µm/sec, KO: 0.26± 0.05 µm/sec, p=0.17, n=4 independent experiments). (**h,i**) Representative images and corresponding kymographs of axons, expressing Mito-mCherry in WT (h) and AP-2 KO (i) neurons. Scale bars, 5µm. (**j**) Bar diagram representing the relative axonal mobility of Mito-mCherry in WT and AP-2µ KO neurons (WT: 7.72±0.94%, KO: 8.59±1.38%, p=0.70, n=3 independent experiments). (**k**) Bar diagram representing the anterograde and retrograde velocity of mitochondria in WT and AP-2µ KO neurons (Retrograde WT: 0.40±0.03 µm/sec, Retrograde KO: 0.31± 0.03 µm/sec, p=0.15; Anterograde WT: 0.36± 0.02 µm/sec, KO: 0.35± 0.01 µm/sec, p=0.64, n=3 independent experiments). (**l,m**) Representative EM snapshots of somata of cultured WT (**l**) and AP-2µ KO (**m**) neurons. Black boxes in (**l**) mark the areas magnified in (**m**). Scale bars, left panel, 1µm, right panel, 500 nm. g, Golgi, n. nucleus, Lys, lysosome. (**n**) Mean number of lysosomes/µm^2 within the soma of AP-2µ KO neurons was unaltered (0.025±0.019) compared to control neurons (0.028±0.015, p=0.84, 9 somata per condition). (**o**) Examples of multilamellar dense structures in synapses of AP-2µ KO neurons. Scale bars, 500nm.

Data in (b) are illustrated as box plots as described in Methods. Data in (d,e,f,g,j,k,n) and all data reported in the text are mean±SEM. n.s., non-significant.

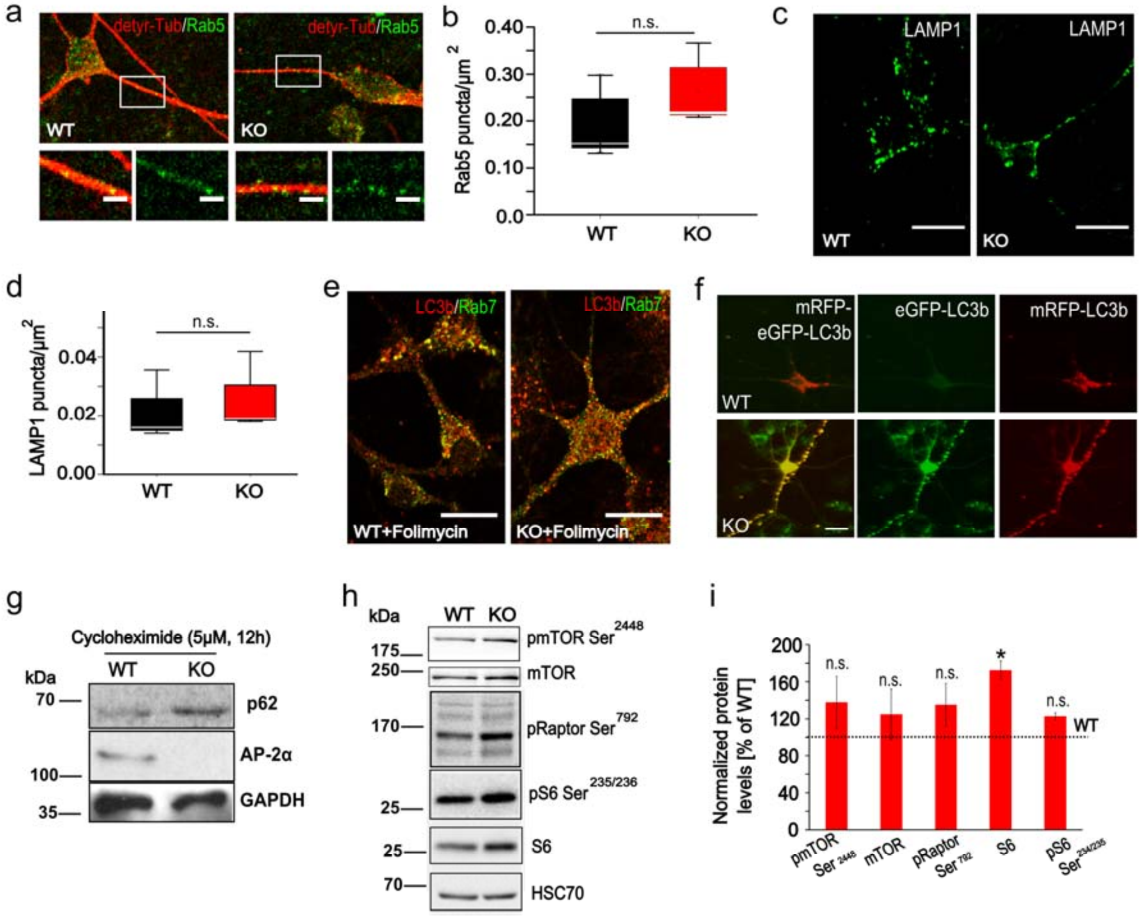

Supplementary Fig. 4. Impaired autophagic flux in the absence of AP-2µ is not a result of decreased mTORC1 signalling. (**a**) Representative confocal images of WT and AP-2µ KO neurons immunostained for Rab5 (green) and detyrosinated tubulin (detyr-Tub, red) to label axonal processes. Scale bars, 5 µm. (**b**) The mean number of Rab5-positive puncta/ 1 μm^2 in AP-2µ KO neurons was unaltered (0.26±0.05) compared to control neurons (0.19±0.05, p=0.4, data are from n=3 independent experiments, 26-27 neurons per condition). (**c**) Representative confocal images of WT and AP-2µ KO neurons immunostained for LAMP1 (green). Scale bars, 20 µm. (**d**) Mean number of LAMP1-positive puncta/ 1 μm^2 in AP-2µ KO neurons was unaltered (0.026±0.007) compared to control neurons (0.022±0.007, p=0.667, n=3, 26-27 neurons per condition). (**e**) Representative confocal images of WT and AP-2µ KO neurons treated with folimycin (20 nM, 4 h) and immunostained for endogenous LC3b (red) and Rab7 (green). Scale

bars, 20 µm. (**f**) Representative epifluorescent images of neurons expressing mRFP-eGFP-LC3 under serum-deprived conditions. Scale bar, 20 µm. (**g**) Immunoblot illustrating the levels of p62 in lysates from cultured WT and AP-2µ KO neurons, treated with cycloheximide. (**h)** Immunoblot illustrating the effect of AP-2µ loss on mTORC1 signalling in brain lysates from p21 neuron-specific AP-2µ KO mice (AP-2$\mu^{lox/lox}$ x Tubα1-Cre). **(i)** Quantification of data shown in (**h**). Protein levels of S6 kinase are slightly increased in AP-2µ KO brain lysates (WT: 1.00±0.01, KO: 1.73±0.09, *p=0.015, n= 3 independent experiments). n.s., non-significant. Protein levels in KO condition were normalized to the WT set to 100%.

Data in (b and d) are illustrated as box plots as described in Methods. Data illustrated in (i) and all data reported in the text are mean±SEM.

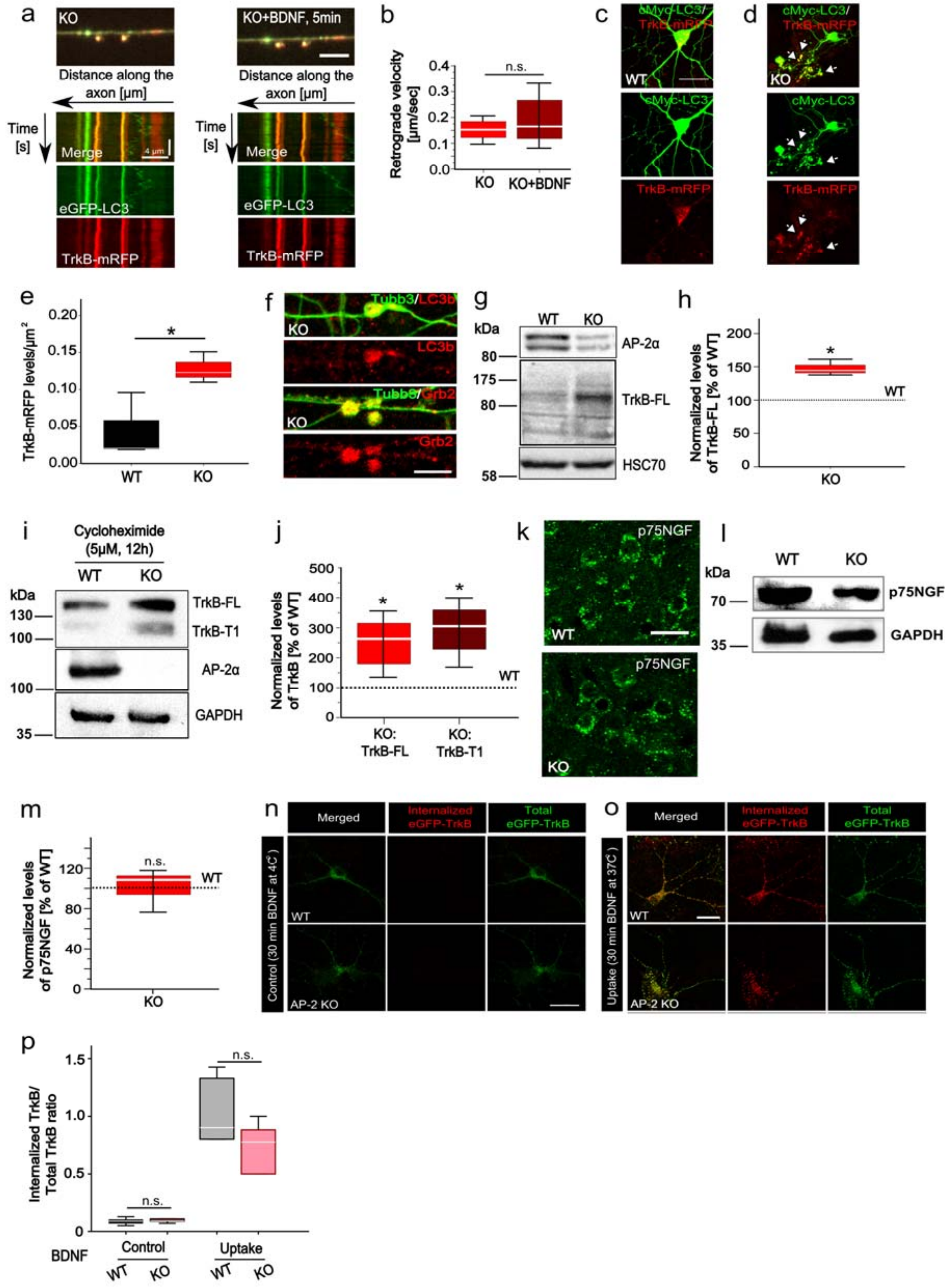

Supplementary Fig. 5. Endocytosis-independent role for AP-2μ in TrkB receptor trafficking. (**a,b**) Retrograde velocity of eGFP-LC3/ TrkB-mRFP-positive carriers in AP-2μ KO neurons is unaltered upon BDNF application (KO: 0.15±0.03 μm/s, KO+BDNF: 0.19±0.07

μm/s; *p=0.65, n=3 independent experiments. (**c,d**) Representative confocal images of WT and AP-2μ KO neurons, expressing cMyc-LC3 (green) and mRFP-TrkB (red). Arrows indicate mRFP-TrkB accumulations within LC3b-positive puncta. Scale bar, 20 μm. (**e**) Loss of AP-2μ leads to accumulation of mRFP-TrkB within LC3b-positive compartments (WT: 0.019±0.025, KO: 0.128±0.012, *p=0.042, n=3, 30 neurons per condition). mRFP-TrkB levels were calculated per 1 μm^2 within LC3-positve area. (**f**) TrkB signalling component Grb2 (growth factor receptor-bound protein 2) accumulates within autophagosome-like structures in AP-2μ KO neurons. Neurons were immunostained for Tubulin beta-3 (Tubb3) and either LC3b or Grb2. (**g,h**) TrkB full length (TrkB-FL) expression levels are significantly upregulated in brain lysates from p21 neuron-specific AP-2μ KO mice compared to controls. TrkB levels in KO were normalized to the WT, set to 100% (KO: 148±7%, *p=0.021, n=3 independent experiments). (**i,j**) Immunoblot analysis of TrkB-FL and its truncated isoform (TrkB-T1) in lysates from cultured WT and AP-2μ KO neurons, treated with cycloheximide. TrkB levels in KO were normalized to the WT, set to 100% (KO TrkB-FL: 248.1±55.5%, *p=0.046, KO TrkB-T1: 293.9±53.5%, *p=0.021, n=3 independent experiments). (**k**) Unaltered levels of p75NGF receptor in AP-2μ KO brains, visualized by immunohistochemistry with p75NGF-specific antibodies. Scale bar, 30 μm. (**l, m**) Immunoblot analysis of p75NGF receptor expression levels in lysates from cultured WT and AP-2 KO neurons. p75NGF levels in KO were normalized to the WT, set to 100% (KO: 103±8.07%, *p=0.38, n=4 independent experiments). (**n,o**) Representative confocal images of eGFP-TrkB-expressing WT and AP-2μ KO neurons treated with 50 ng/ml BDNF and incubated with GFP antibody at 4 C° (control, **n**) or 37 C° to allow endocytosis (uptake, **o**). Scale bars, 50 μm. (**p**) BDNF-induced endocytosis of TrkB is independent of AP-2μ. Control (4C°) WT versus KO, p=0.668; Uptake (37°C) WT versus KO, p=0.098, n=5 independent experiments.

Data in (b,e,h,j,m) and (p) are illustrated as box plots as described in Methods. All data reported in the text are mean±SEM.

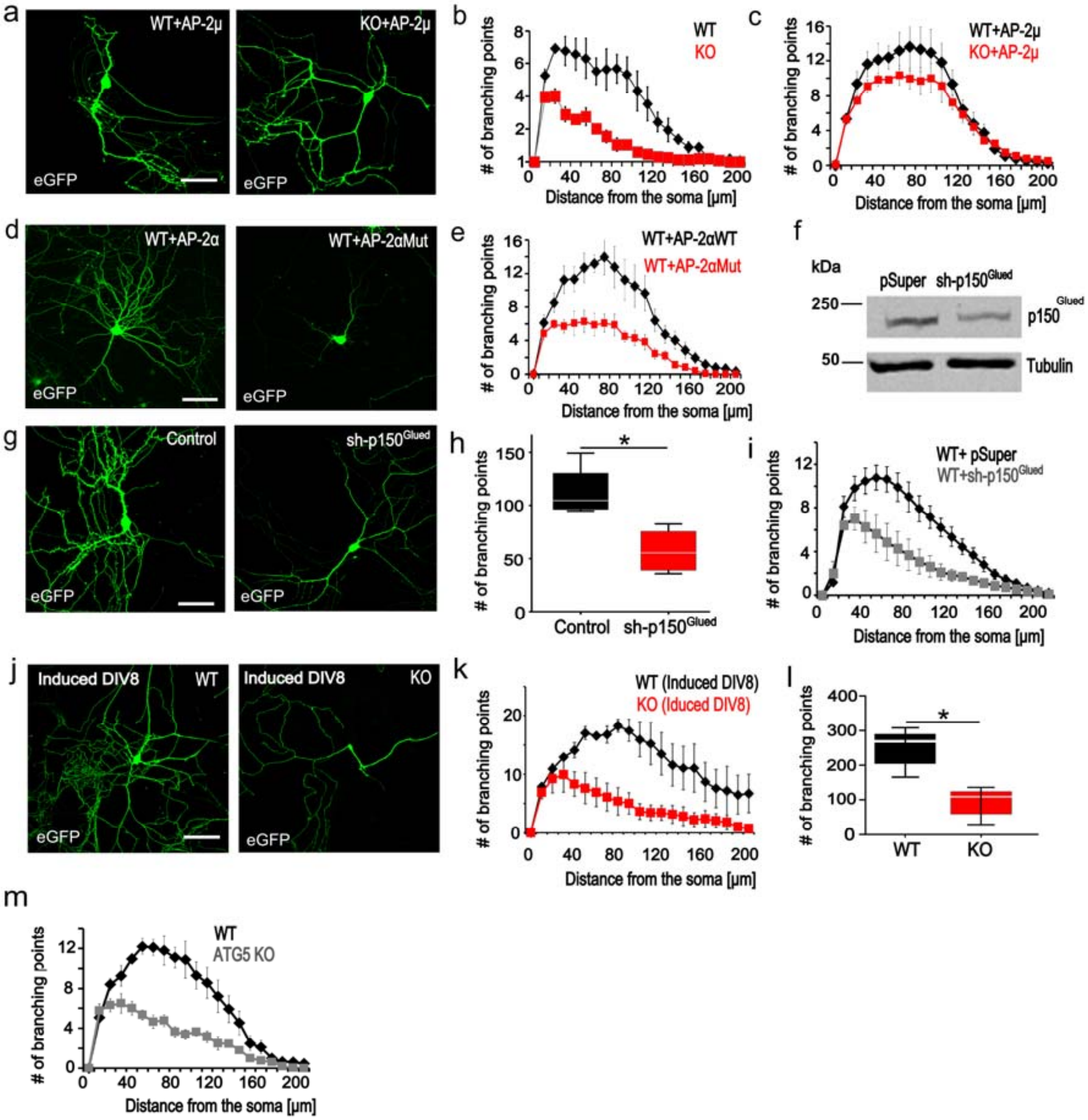

Supplementary Fig. 6. Autophagosomal trafficking via AP-2 mediates neuronal complexity. **(a)** eGFP-expressing WT and AP-2μ KO neurons transfected with AP-2μ. Scale bar, 40 μm. (**b**) Sholl analysis of cultured WT and AP-2μ KO neurons reveals the decrease in neuronal complexity upon loss of AP-2μ. (**c**) Sholl analysis of cultured WT and AP-2μ KO neurons reveals the rescue of neuronal complexity in AP-2μ KO neurons upon AP-2μ re-expression. (**d**) Control neurons transfected with eGFP and co-transfected with HA-tagged WT AP-2α_A (HA-AP-2α_AWT) or LC3-binding deficient mutant AP-2α_A (HA-AP-2α_AMut). Scale bar, 40 μm. (**e**) Sholl analysis of neurons, expressing wildtype AP-2α_A (AP-2α_AWT, black) or LC3-binding

deficient mutant AP-2α_A (AP-2α_AMut, red). (**f**) p150^{Glued} levels in cultured rat neurons, nucleofected on DIV0 with either pSuper control or pSuper encoding shRNA against p150^{Glued} and analyzed 48 h postransfection by immunoblotting. **(g)** eGFP-expressing rat neurons transfected on DIV7 with either pSuper control (WT+pSuper) or with shRNA against p150^{Glued} (WT+ shRNA p150^{Glued}). Scale bar, 40 µm. (**h)** Mean number of branching points is severely decreased in WT rat neurons expressing p150^{Glued} shRNA (22.05±11.03) compared to control (113.29±12.52, *p=0.015, 79-93 neurons per condition, n=4 experiments). **(i)** Sholl analysis of cultured rat neurons 72h posttransfection with either pSuper control (black) or pSuper encoding shRNA against p150^{Glued}(gray). (**j-l)** AP-2µ deletion induced by application of tamoxifen at DIV8 impairs the neuronal complexity of mature DIV20 neurons. (**j**) eGFP-expressing WT and AP-2µ KO neurons at DIV20. Scale bar, 40 µm. **(k**) Sholl analysis of cultured WT and AP-2µ KO neurons reveals a decrease in neuronal complexity upon induction of AP-2µ deletion at DIV8. (**l**) Decreased branching complexity in neurons treated by tamoxifen at DIV8 (91.33±32.37), but not in ethanol-treated controls (246.90±45.54, *p=0.043, 28-29 neurons per condition from 3 independent experiments). **(m)** Sholl analysis of cultured neurons from ATG5$^{lox/lox}$:CAG-iCre mice, treated either with ethanol (WT) or tamoxifen (ATG5 KO).

Sholl analysis was performed by counting the number of intersections with concentric rings radiating every 10 µm from the soma center. Data in (h,l) are illustrated as box plots as described in Materials and Methods. Data in (b,c,e,i,k,m) and all data reported in the text are mean±SEM.

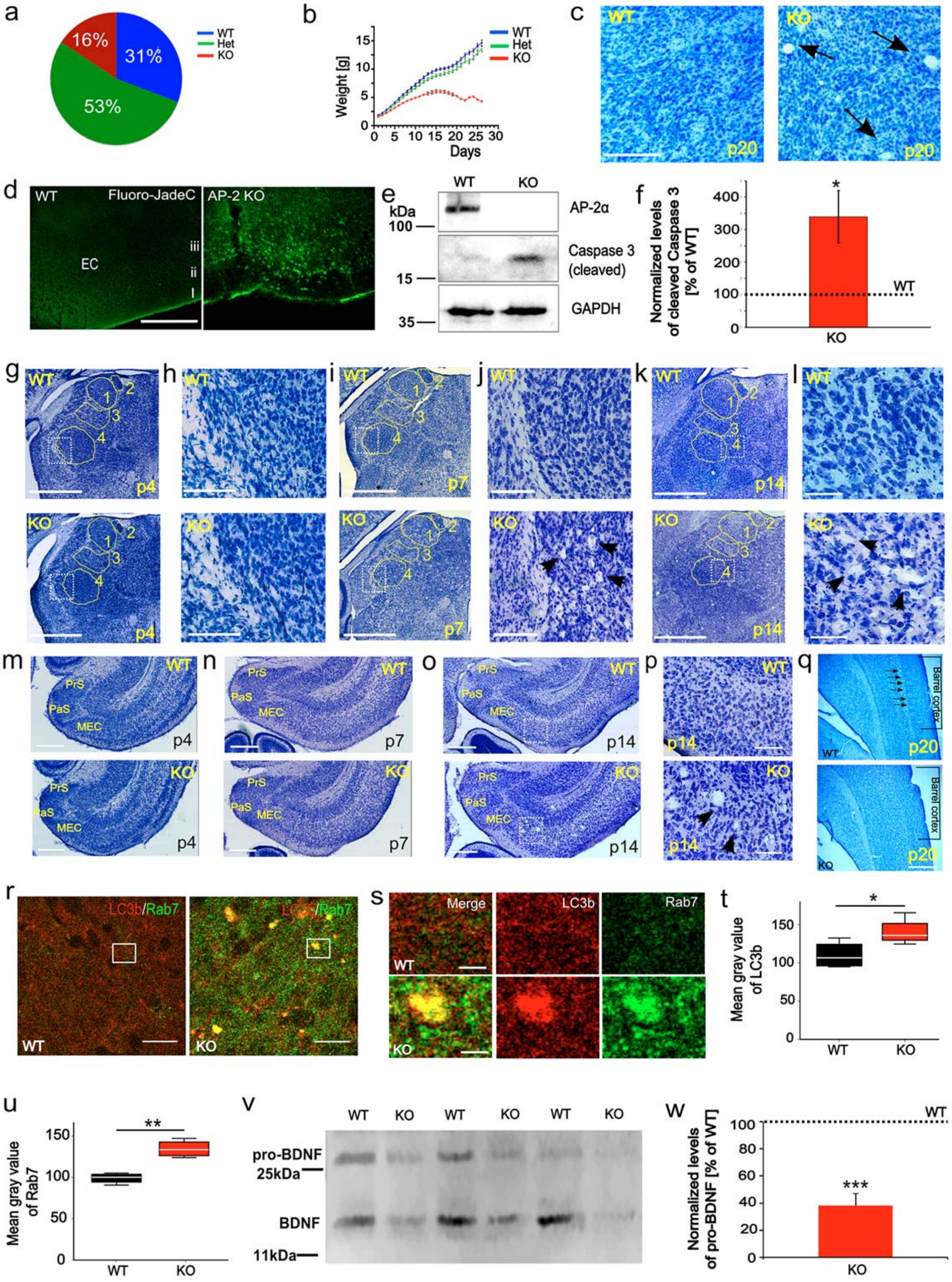
a
16%
31%
53%
WT
Het
KO
b
Weight [g]
Days
WT
Het
KO
c
WT
KO
p20
d
WT
Fluoro-JadeC
AP-2 KO
EC
iii
ii
I
e
WT
KO
kDa
100
15
35
AP-2α
Caspase 3
(cleaved)
GAPDH
f
Normalized levels
of cleaved Caspase 3
[% of WT]
WT
KO
g h i j k l
WT
KO
p4
p7
p14
m n o p q
PrS
PaS
MEC
Barrel cortex
p20
r
LC3b/Rab7
WT
KO
s
Merge
LC3b
Rab7
t
Mean gray value
of LC3b
u
Mean gray value
of Rab7
v
pro-BDNF
25kDa
BDNF
11kDa
w
Normalized levels
of pro-BDNF [% of WT]

Supplementary Fig. 7. Neurodegeneration in mice lacking neuronal AP-2µ. (a) Percentage of genotypes in litters from AP-2$^{lox/lox}$:Tub α1-Cre crosses (AP-2$^{lox/lox}$:Tubα1-Cre (KO): 16%; AP-2$^{lox/lox}$:Tubα1-Cre (WT): 31% and AP-2$^{lox/wt}$:Tubα1-Cre (Het): 53%; p< 0.0011). **(b)** Growth curves of AP-2µ KO mice and their littermate controls (KO: n= 14, WT and Het: n= 21 animals). (**c**) Loss of neuronal AP-2µ causes thalamic microvacuolation (indicated by arrows). Scale bar, 250µm. (**d**) Severe neurodegeneration of AP-2µ KO brains, detected by Fluoro-Jade staining. Scale bar, 400 µm. (**e,f**) Immunoblot analysis representing the activation of caspase 3 in lysates from cultured DIV14-17 WT and AP-2µ KO neurons. Activated caspase 3 levels in KO were normalized to the WT, set to 100% (KO: 339.7±80%, *p=0.014, n=5 independent experiments). (**g-l**) Temporal progression of thalamic neurodegeneration in AP-2µ KO mice, captured by Nissl-staining of brains at p4 (**g,h**), p7 (**i,j**) and p14 (**k,l**). White rectangles in (**g**) (**i**) and (**k**) mark the areas magnified in (**h**), (**j**) and (**l**), accordingly. Black arrows in (**l**) mark the spongiform neurodegeneration. Numbers indicate thalamic nuclei: 1, anteroventral, 2, anterodorsal, 3, ventrolateral, 4, posterior. (**m-p**) Temporal progression of medial entorhinal cortex (MEC) degeneration in AP-2µ KO mice, captured by Nissl-staining of brains at p4 (**m**), p7 (**n**) and p14 (**o,p**). Black arrows in (**p**) mark the spongiform neurodegeneration. Scale bars, (g,i) 1000µm, (h, j) 150 µm, (k) 800µm, (l) 100 µm, (m,n) 300µm, (o) 400µm, (p) 80 µm. (**q**) Loss of barrel compartments in the somatosensory cortex of AP-2µ KO mice, revealed by Nissl-staining. Scale bar, 200 µm. (**r**) WT and AP-2µ KO brain sections, immunostained for LC3b and Rab7. White boxes indicate areas magnified in (**s**). Scale bars: (**r**) 20 µm, (**s**) 5 µm. (**t, u**) Mean intensity of LC3b (WT: 110.1±8.7, KO: 140.5±8.8, *p=0.049, n=4) (**u**) and Rab7 (WT: 99.1±3.1, KO: 134.5±5.2, **p=0.001, n=4) (**u**) in AP-2µ deficient brains. (**v,w**) pro-BDNF levels in WT or AP-2µ KO brain lysates visualized by immunoblotting on the membrane presented in Fig. 7b. Pro-BNDF levels in WT brains were set to 100% (n=3 mice per genotype, *p<0.000).

Data in (t,u) are illustrated as box plots. Data in (a,b,f,w) and all data reported in the text are mean±SEM.

Fig. 2a

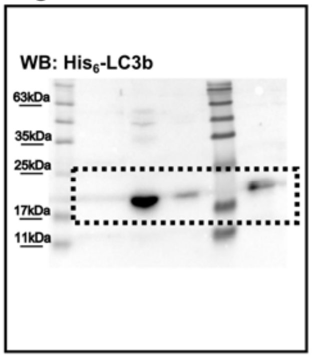

Fig. 2b

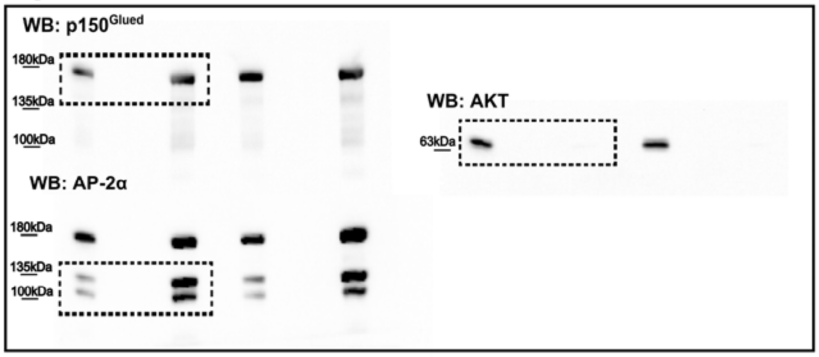

Fig. 2c

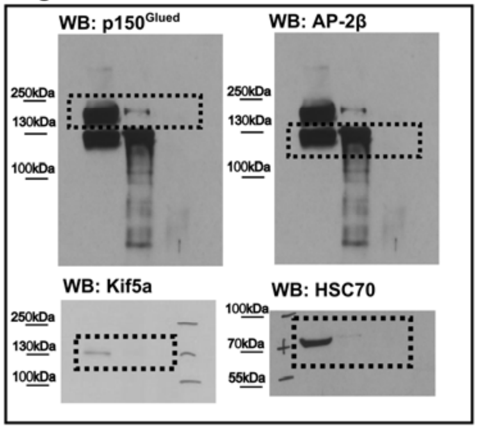

Fig. 2d

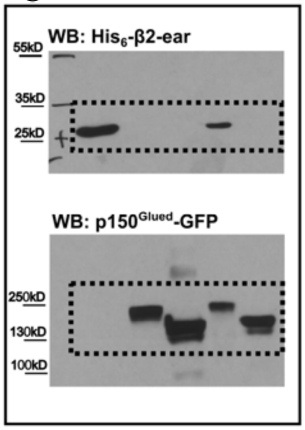

Fig. 2e

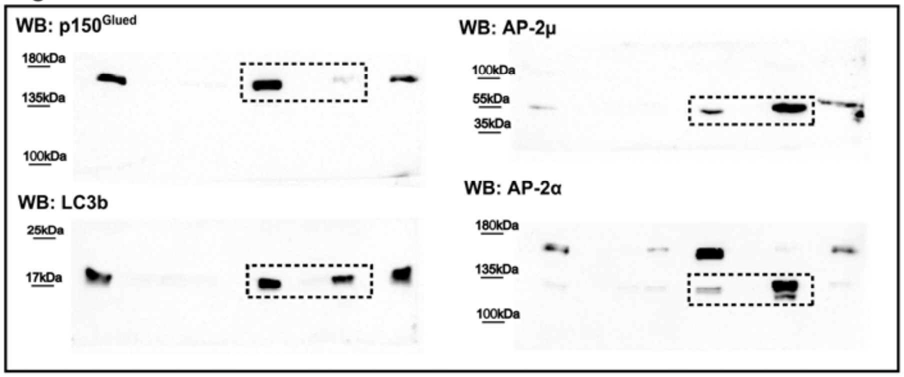

Fig. 7b, Fig. S7v

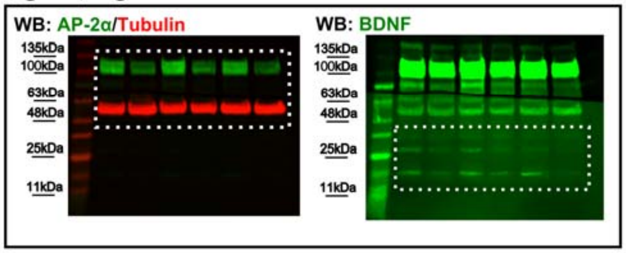

Fig. S2b

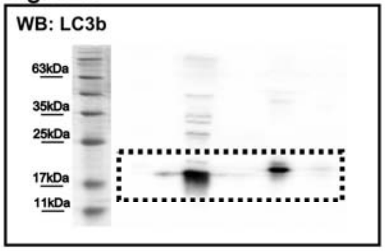

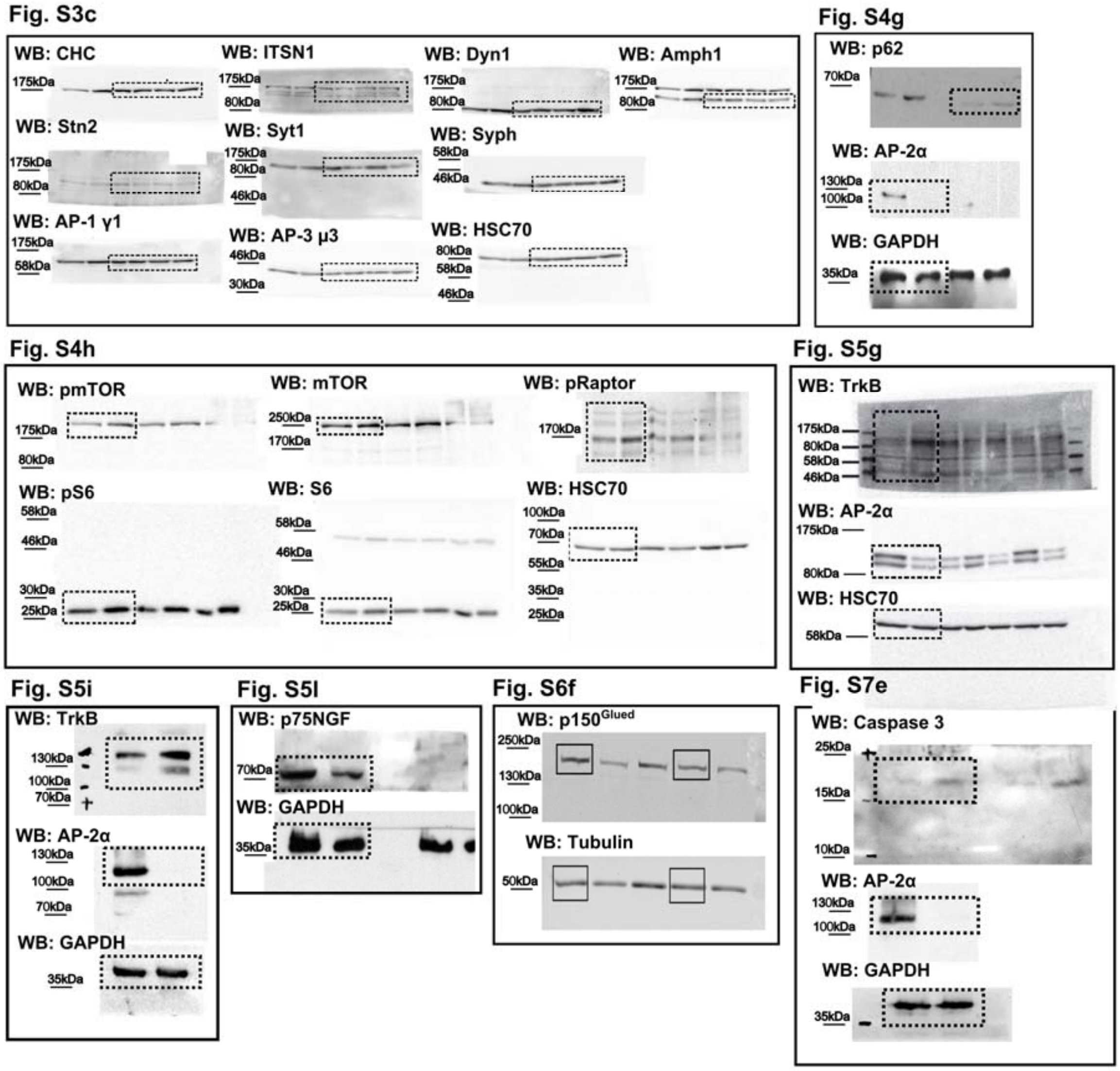

Supplementary Fig. 8. Full-size blots corresponding to cropped imaged presented in the manuscript.
